# Moisture sorption isotherm and changes in physico‐mechanical properties of films produced from waste flour and their application on preservation quality of fresh strawberry

**DOI:** 10.1002/fsn3.589

**Published:** 2018-02-06

**Authors:** Rattana Muangrat, Chanida Nuankham

**Affiliations:** ^1^ Division of Food Process Engineering Faculty of Agro‐Industry Chiang Mai University Chiang Mai Thailand; ^2^ Division of Food Science and Technology Faculty of Agro‐Industry Chiang Mai University Chiang Mai Thailand

**Keywords:** films, moisture sorption isotherm, waste flour

## Abstract

Waste flour from the noodle industry was used to produce films, which were plasticized with 40% w/w glycerol:sorbitol at 2:1, 3:1, and 4:1 w/w and formulated with 200 ppm potassium sorbate. Henderson's equation was found to be the best estimator for moisture sorption isotherm of the films stored at 5, 25, and 45°C, and then, equilibrated at 0.11, 0.23, 0.32, 0.43, 0.58, 0.64, 0.76, 0.84, and 0.93 water activity. Developed flour films (plasticized with 2:1 w/w glycerol:sorbitol/formulated with 20% w/w potassium sorbate), with the best mechanical properties (tensile strength of 1.05 MPa; elongation at break of 73.01%), were used to cover fresh strawberries on a polystyrene foam tray. It was found that higher average phenolic contents, antioxidant activity, and firmness were found in strawberries wrapped in plasticized/formulated films, when compared against both films without potassium sorbate and without film (control). Furthermore, a lower average total microorganism count was found for fresh strawberries wrapped in the plasticized/formulated films, when compared with films without potassium sorbate.

## 1 INTRODUCTION

Rice is one of the most economically important plants worldwide (Majzoobi, Pesaran, Mesbahi, Golmakani, & Farahnaky, 2015). A vast range of processed rice products, such as flour products made from rice and various types of noodles, snacks, and convenience foods, are manufactured to meet diverse consumer demands. Many rice products are made from rice flour, a starchy, low‐cost rice that is broken during the milling process (Majzoobi et al., 2015). Broken rice contains 64.38%–80.84% carbohydrate, 7.76%–8.26% protein, 0.39%–1.49% fat, 0.31%–0.6% ash, and 9.60%–14.37% moisture (Hou & Zhu, 2010; Majzoobi et al., 2015). Rice flour is a natural material, suitable for making films, due to the properties associated with the amylose and amylopectin molecules. The binding that occurs between the amylose and amylopectin yields a strong film, in which the starch forms a continuous network.

According to the study by Dias, Müller, Larotonda, and Laurindo (2010), rice flour films had a higher elongation at break but a lower tensile strength than rice starch films, when the same contents of plasticizer (glycerol and sorbitol) were used. Moreover, studying the properties of rice starch–chitosan films, combined with the antimicrobial agent triclosan, and plasticized with 30% glycerol, Bourtoom and Chinnan (2008) found that the films had a higher elongation at break and water vapor transmission rate (WVTR) than those prepared from starch and celluloses. In addition, Laohakunjit and Noomhorm (2004), Borges, Romani, Cortez‐Vega, and Martins (2015) and Majzoobi et al. (2015) studied the attributes of rice starch films and noted that the incorporation of plasticizers (glycerol and sorbitol) increased the elongation at break and WVTR.

Therefore, this research focused on developing films made from natural biopolymers, using the waste flour obtained from the noodle industry, to increase its value. Flour films plasticized with glycerol and sorbitol were produced, and the changes in their equilibrium moisture contents were investigated at various temperatures and water activities. Additionally, their physico‐mechanical properties (water vapor permeability [WVP], tensile strength, and elongation at break) were examined during storage at 5, 25, and 45°C, 58% relative humidity (RH), for 30 days. Finally, films displaying the most desirable physico‐mechanical properties were applied on fresh strawberries (on a polystyrene foam tray), and their effects on microbial control were examined during storage at 5°C, 95% RH, for 9 days.

## 2 MATERIALS AND METHODS

### 2.1 Materials

This study used the leftover (broken) flour from the noodle steaming process conducted at Iisariyaphon Limited Partnership noodle factory in Chiang Mai, Thailand. The material was stored in tightly sealed plastic bags at 4°C.

### 2.2 Preparation and sorption isotherms of the flour films

Films were produced, by mixing the waste flour with 75% distilled water (w/v), to make a starch solution that was then plasticized with 40% w/w glycerol:sorbitol at 2:1, 3:1, and 4:1 w/w, respectively, based on the dry weight of the waste flour. Two‐hundred ppm potassium sorbate was added to an independent series of each film to prevent fungi and mold growth. The starch solution was heated at 90°C for 15 min with stirring. The solution was then cast onto an acrylic sheet (25 × 25 × 0.10 cm, width × length × depth), used as a mold. The films were dried at 55°C for 24 hr, before they were cut into pieces (4 × 4 cm), each weighing approximately 1 g. The pieces were predried over P_2_O_5_ solution (65% RH) in a sealed container for 7 days and then placed over saturated salt solutions (LiCl, CH_3_COOK, MgCl_2_·6H_2_O, K_2_CO_3_, NaBr, NaNO_2_, NaCl, KCl, and KNO_3_), having water activities of about 0.11, 0.23, 0.33, 0.43, 0.58, 0.64, 0.76, 0.84, and 0.93, respectively, in sealed Erlenmeyer flasks and stored in an incubator at 5, 25, and 45°C. The samples were weighed every 3 days until a constant weight was attained (±0.001 g). The samples were then oven‐dried at 105°C for 24 hr to obtain the moisture contents. The moisture sorption data were fitted to five mathematic equations including Henderson's (1952), Smith's (1947), Halsey's (1948), Oswin's (1946), and Guggenheim–Anderson–de Boer's (GAB) (Van Den Berg, 1985) as shown in Equations (1), (2), (3), (4), (5), respectively.

Henderson's equation:

(1) $$M_{w}={\left[\frac{\ln\left(1-a_{w}\right)}{-A}\right]}^{1/B}$$

Smith's equation:

(2) $$M_{w}=A+B\ln\left(1-a_{w}\right)$$

Halsey's equation:

(3) $$M_{w}={\left[-\frac{A}{\ln a_{w}}\right]}^{1/B}$$

Oswin's equation:

(4) $$M_{w}=A{\left[\frac{a_{w}}{1-a_{w}}\right]}^{B}$$

GAB equation:

(5) $$M_{w}=\frac{M_{0}Cka_{w}}{\left(1-ka_{w}\right)\left(1-ka_{w}+Cka_{w}\right)}$$

where *M*_w_ is the equilibrium moisture content (g water/100 g dry matter), *M*_0_ is the GAB monolayer moisture (g water/100 g dry film), *a*_w_ is water activity, and *A*,* B*,* C,* and *k*, are constants.

### 2.3 Properties of the films

The physico‐mechanical (tensile strength, elongation at break, and WVP) properties of the aforementioned film specimens stored at 5, 25, and 45°C, at 58% RH, were analyzed every 3 days, for 30 days.

#### 2.3.1 Tensile strength and elongation at break

Before analysis, the films were cut into strips (25 × 150 mm) and then equilibrated at 65 ± 2% RH and 27 ± 2°C, using an HWS Intelligent Constant Temperature and Humidity Machine (Ningbo, China). Tensile strength and elongation at break were measured, using an Instron Universal Testing Machine Model 1000 (H1K‐S, UK), according to the American Standard for Testing and Materials (ASTM D882‐80a, 1995), at a crosshead speed of 25 mm/min and an initial gauge length of 100 mm. Tensile strength was calculated by dividing the maximum force by the initial specimen cross‐sectional area according to the following Equation (6). Elongation at break (%) was calculated using Equation (7):

(6) $$\text{Tensile strength (MPa)}=\frac{F_{\max}}{A_{0}}$$

where *F*_max_ is maximum force applied (*N*) and *A*_0_ is initial specimen cross‐sectional area (m^2^)

(7) $$\text{Elongation at break}\;\left(\%\right)=100\times \frac{(\ell_{\mathrm{after}}-\ell_{\mathrm{before}})}{\ell_{\mathrm{before}}}$$

where ℓ is the distance between grips holding the specimen before or after the break of the specimen. Mean values of tensile strength and elongation at break were calculated from quintuplicate measurements.

#### 2.3.2 Water vapor permeability

The WVP of the films was measured gravimetrically according to the method reported by Klangmuang and Sothornvit (2016). The film specimens stored at 5, 25, and 45°C at 58% RH were equilibrated at 50% RH and 25°C for at least 24 hr before testing. The test cup (4.50 × 4.23 × 4.00 cm, outer diameter × inner diameter × depth) was filled with 20 ml distilled water. The cup was then closed by the films and was placed into a cabinet at 25°C, 50% RH. The cup had an exposed film area of 14.05 cm^2^. The change in weight of the cup was recorded (±0.001 g) at 1‐hr intervals until constant weight. The weight loss of the cup was plotted as a function of time, to estimate the slope, by a linear equation (the correlation coefficient, *R* ^2^ was .99). Once the slope was obtained, the WVTR was calculated (Equation (8)) followed by the WVP (Equation (9)).

(8) $$\text{WVTR}=\frac{m}{t\times A}$$

(9) $$\text{WVP}=\frac{\text{WVTR}\times L}{\Delta p}$$

where WVTR is the water vapor transmission rate (g/m^2^ day), WVP is the water vapor permeability (g mm/m^2^ day kPa), *m* is weight (g), *t* is time (day), *m*/*t* is slope of the straight line (weight loss per unit time) (g/day), *L* is the mean film thickness (mm), *A* is test area (m^2^), and ∆*p* is the water vapor partial pressure difference across the films (kPa).

### 2.4 Water solubility

Film sample, 20 × 20 mm, was dried at 105°C for 24 hr to determine the initial dry matter (*W*_i_). The film sample was immersed in 30 ml of distilled water, slowly agitated and then kept at 25°C for 24 hr. The insoluble portion of the film sample was filtered and dried at 105°C for 24 hr to determine the final weight of dry matter of film sample (*W*_f_). The water solubility measurements were replicated three times for each film sample. The water solubility of film was calculated as the percentage of dry matter of the solubilized film after immersion in water at 25°C for 24 hr and was determined using Equation (10).

(10) $$\text{Water solubility}\;\left(\%\right)=\frac{W_{i}-W_{f}}{W_{i}}\times 100\%$$

### 2.5 Determination of abilities of flour films with added potassium sorbate to preserve strawberries during storage

The films mentioned in Section 2.3 were used to wrap fresh strawberries contained on a polystyrene foam tray that were then stored at 5°C, 90% RH, for 9 days. The quality of the fresh strawberries (texture, total microorganisms including yeast and mold, measured by total plate count method, phenolic compounds, antioxidant activity by DPPH assay, and total soluble solids [°Brix]) was analyzed at 0, 3, 6, and 9 days. Total phenolic content expressed as mg gallic acid equivalent/100 g fresh strawberries was estimated by Folin–Ciocalteu's method (Benzie & Strain, 1996). DPPH scavenging activity was expressed as mg Trolox equivalent/g of fresh strawberries using the standard Trolox curve (Brand‐Williams, Cuvelier, & Berset, 1995).

### 2.6 Statistical analysis

Based on a completely randomized design, with three repeats, all data were statistically evaluated by analysis of variance (ANOVA) followed by Duncan's multiple range test at 95% reliability to compare the means.

## 3 RESULTS AND DISCUSSION

### 3.1 Moisture sorption isotherms of the flour films

**Figure 1:**
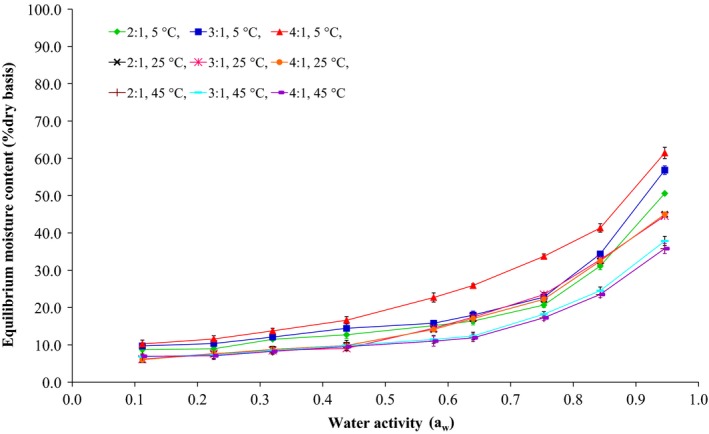
Equilibrium moisture content of the films produced from waste flour

The moisture sorption curves of waste flour (75% w/v) films, plasticized with 40% w/w glycerol:sorbitol at 2:1, 3:1, and 4:1 w/w, and containing 200 ppm potassium sorbate are presented in Figure 1. The results revealed that the equilibrium moisture content of the films increased with increasing water activity but it decreased with increasing temperature. The sigmoid‐shaped curves shown in the graph represent the sorption isotherm of the films according to Brunauer, Emmett, and Teller (1938). The increase in the equilibrium moisture content can be seen from the linear graphs, which possibly bend to the water activity of 1, which is characteristic of foods with high starch content. Furthermore, the plasticizers incorporated into the films had more desiccant abilities than the flour at all water activities. Previous studies have shown that the desiccant abilities of flour films differ depending on the type of plasticizer used for a set flour weight (Borges et al., 2015; de la Cruz, Torres, & Martín‐Polo, 2001; Kibar & Us, 2013; Turhan & Şahbaz, 2004). In this study, the films prepared at 4:1 w/w glycerol:sorbitol were found to have stronger desiccant abilities than the films with 3:1 and 2:1 w/w glycerol:sorbitol, respectively (Figure 1). Numerous researchers (Borges et al., 2015; Jamali et al., 2006; Mali, Grossmann, García, Martino, & Zaritzky, 2006; Souza et al., 2012; Zhang & Han, 2008) have reported that glycerol‐plasticized films have significantly greater desiccant abilities than other plasticizers. This is because glycerol is a highly hydrophilic plasticizer that forms hydrogen bonds with water molecules via its hydroxyl groups.

The sorption isotherm data of the waste flour films (Figure 1) were fitted to the mathematic equation models of Henderson, Smith, Halsey, Oswin, and GAB. The model constants, adjusted coefficient of determinations ($R_{\mathrm{adj}}^{2}$ ), and root mean square error (RMSE) were calculated for the films with 2:1, 3:1, and 4:1 w/w glycerol:sorbitol, respectively. Based on the findings, the humidity changes in the films kept at the various temperatures and water activities were most adequately described by Henderson's equation because this model had the highest $R_{\mathrm{adj}}^{2}$ value and the lowest RMSE value, among those evaluated, as shown in Table 1.

**Table 1:** Model constants*,* adjusted coefficient of determination ($R_{\mathrm{adj}}^{2}$ ), and root mean square error (RMSE) of the best estimator (modified Henderson model) for moisture sorption isotherm of films produced from waste flour, with 40% plasticizers (2:1, 3:1, and 4:1 w/w glycerol:sorbitol ratio) and 200 ppm potassium sorbate

Sample	Temperature (°C)	Model	Equation parameters	*R* ^2^	RMSE
Films produced from waste flour, with 40% plasticizers (2:1 w/w glycerol:sorbitol ratio)					
	5	Modified Henderson	*A *=* *0.0049; *B *=* *1.7529	.9513	0.0556
	25	Modified Henderson	*A *=* *0.0061; *B *=* *1.7777	.9529	0.0547
	45	Modified Henderson	*A *=* *0.0073; *B *=* *1.7997	.9524	0.0540
Films produced from waste flour, with 40% plasticizers (3:1 w/w glycerol:sorbitol ratio)					
	5	Modified Henderson	*A *=* *0.0040; *B *=* *1.7712	.9525	0.0550
	25	Modified Henderson	*A *=* *0.0060; *B *=* *1.7534	.9514	0.0556
	45	Modified Henderson	*A *=* *0.0085; *B *=* *1.7379	.9504	0.0561
Films produced from waste flour, with 40% plasticizers (4:1 w/w glycerol:sorbitol ratio)					
	5	Modified Henderson	*A *=* *0.0043; *B *=* *1.7311	.9500	0.0564
	25	Modified Henderson	*A *=* *0.0068; *B *=* *1.6940	.9475	0.0578
	45	Modified Henderson	*A *=* *0.0101; *B *=* *1.6622	.9452	0.0590

### 3.2 Properties of flour films during storage

**Figure 2:**
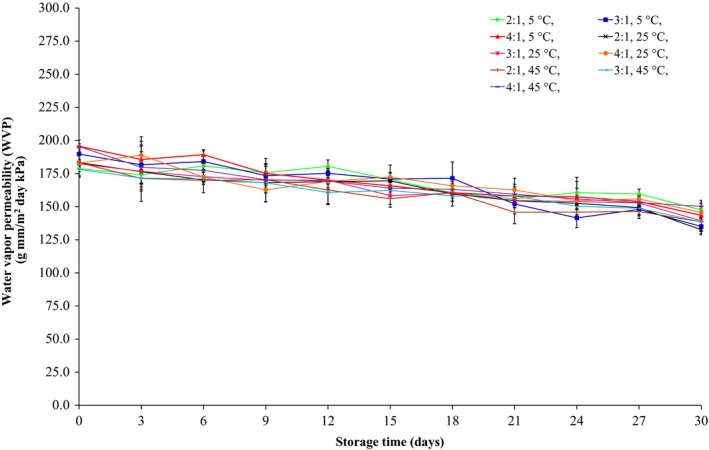
The water vapor permeability of the films produced from waste flour, with 40% glycerol and sorbitol (2:1, 3:1, and 4:1 w/w) and 200 ppm potassium sorbate, stored at 5, 25, and 45°C

**Figure 3:**
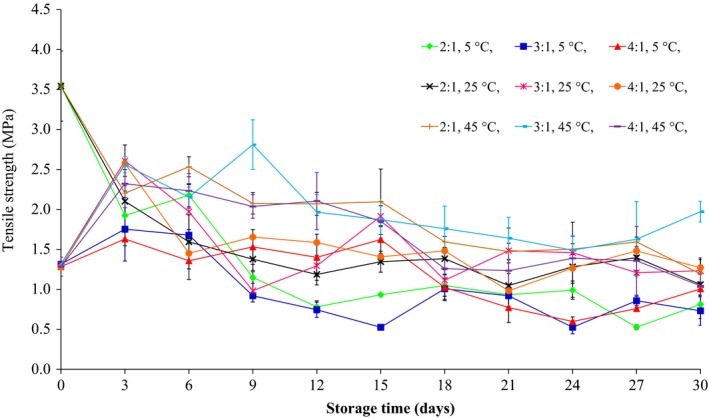
The tensile strength of the films produced from waste flour, with 40% glycerol and sorbitol (2:1, 3:1, and 4:1 w/w) and 200 ppm potassium sorbate, stored at 5, 25, and 45°C

**Figure 4:**
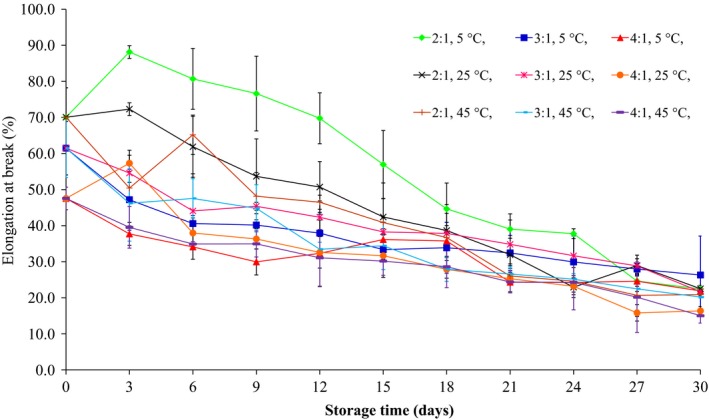
The elongation at break of the films produced from waste flour, with 40% glycerol and sorbitol (2:1, 3:1, and 4:1 w/w) and 200 ppm potassium sorbate, stored at 5, 25, and 45°C

The effects of storage time and glycerol:sorbitol w/w ratio on the WVP, tensile strength, and elongation at break of films are shown in Figures 2, 3, 4, respectively. The WVP, tensile strength, and elongation at break of the films plasticized with 40% glycerol and sorbitol (2:1, 3:1, and 4:1 w/w) and 200 ppm potassium sorbate decreased significantly (*p* ≤ .05) during storage at 5, 25, and 45°C, at average RH of 58%, for 30 days. Irrespective of the glycerol:sorbitol w/w ratio, a difference in WVP values was also related to the glycerol:sorbitol w/w ratio, with the higher plasticizer content yielding higher WVP values. The tensile strength and elongation at break of flour‐based films decreased as plasticizer concentration increased. However, the tensile values of films were fluctuated throughout 15 days and were gradually stable after 18 days of storage period. Additionally, the films stored at 45°C had a lower tensile strength (Figure 3) and elongation at break (Figure 4) than those stored at 5 and 25°C. Furthermore, at 24 days storage, at 5 and 25°C, the elongation at break of the films plasticized with 2:1 w/w glycerol:sorbitol was higher than that obtained for 3:1 and 4:1 w/w glycerol:sorbitol, respectively; however, their elongation values were not statistically different (*p* > .05) during storage from 27 to 30 days.

Butler, Vergano, Testin, Bunn, and Wiles (1996) reported that the WVP and tensile strength of chitosan films plasticized with glycerin and containing triclosan decreased throughout 12 weeks of storage. Furthermore, Osés, Fernández‐Pan, Mendoza, and Maté (2009) studied the effect of storage time on the mechanical properties of whey protein isolate‐based films plasticized with glycerol and sorbitol stored at room temperature, at 50% and 75% RH, for 30 weeks. The authors noted that the storage time did not affect the tensile strength and the elongation values at break of the films plasticized with glycerol, whereas the tensile strength of the films plasticized with sorbitol increased with time due to sorbitol crystallization.

In the current study, the WVP, tensile strength, and elongation at break of all film specimens decreased after storage at 5, 25, and 45°C for 30 days. These changes could possibly be attributed to the instability of the plasticizers (glycerol and/or sorbitol) due to plasticizer migration from the film matrix to the surface and the surrounding environment during storage time (Heikkinen et al., 2014; Osés et al., 2009). Storage time decreased the WVP and the elongation at break of all films. It is indicated that plasticization and free volume in the film network were decreased after glycerol migration out of the film matrix (Heikkinen et al., 2014; Mikkonen et al., 2009).

Several previous studies have shown that increasing glycerol content caused a decrease in glass transition temperature of film resulting in the decrease in tensile strength but the increase in the elongation at break (García, Martino, & Zaritzky, 2000; Mali et al., 2006; Rindlav‐Westling, Stading, Hermansson, & Gatenholm, 1998). Enrione, Osorio, Pedreschi, and Hill (2010) reported that the glass transition temperature of the starch from corn and rice decreased from 120°C to 60 and 40°C after the addition of 10% and 20% glycerol, respectively. Furthermore, glycerol can decrease crystallization of films due to the decreased glass transition temperature. The crystallization rate and glass transition temperature of the films were not measured in the current study. However, Mali et al. (2006), studying the effect of storage time on various properties of starch films produced from corn, cassava, and sweet potato, found that in the absence of glycerol, the three types of films had higher glass transition temperatures than those with added glycerol. Also, after the three types of films were stored at 20°C for 90 days, the glass transition temperatures of the three types of films with or without glycerol decreased. Besides, without glycerol, there was more crystallization in the films compared to those with glycerol, which caused the WVP and the elongation at break to decrease and caused the tensile strength to increase.

### 3.3 Effect of concentration of potassium sorbate on the properties of the flour films used to preserve strawberries

From the results of physico‐mechanical properties of produced films as shown in Figures 2, 3, 4, the flour films (75% w/v) with glycerol and sorbitol (2:1 w/w) (40%, weight per dry weight of the waste flour) which kept at storage temperature range of 5–25°C (58% RH) showed high potential to improve mechanical properties (e.g., low tensile strength and high elongation at break) for wrapping fresh fruits. Thus, the films were formulated with 10%, 20%, and 30% potassium sorbate (weight per weight of the flour), and the properties of the films produced are shown in Table 2. It was found that the tensile strength decreased but the elongation at break increased with increasing potassium sorbate content. This is because the added potassium sorbate can form bonds with the hydroxyl groups of the flour and the result caused a decrease in intermolecular forces in flour films and an increase in the biopolymer mobility, which could decrease the tensile strength and increase the elongation at break (Barzegar, Azizi, Barzegar, & Hamidi‐Esfahani, 2014; Cagri, Ustunol, & Ryser, 2001).

**Table 2:** Properties of the waste flour films produced with glycerol and sorbitol plasticizers (2:1 w/w) (40%, weight per dry weight of the waste flour), with or without potassium sorbate

Properties of flour films	Amount of potassium sorbate (%)			
	0 (Control)	10	20	30
Water solubility (%)	13.07 ± 0.98^a^	11.32 ± 0.87^ab^	12.15 ± 0.83a^b^	10.81 ± 1.09^c^
Water vapor permeability (g mm/m^2^ day kPa) (25°C, 50% RH)	174.07 ± 10.58^b^	189.30 ± 3.77^a^	191.83 ± 1.65^a^	195.75 ± 4.77^a^
Tensile strength (MPa)	1.53 ± 0.15^a^	1.26 ± 0.14^ab^	1.05 ± 0.09^bc^	0.98 ± 0.13^c^
Elongation at break (%)	63.61 ± 0.96^b^	65.56 ± 2.96^ab^	73.01 ± 2.61^a^	62.04 ± 7.59^b^

Different superscript letters (a–c) used in the same row indicate statistically different values (*p* ≤ .05).

The water solubility decreased with increasing potassium sorbate content. Adding potassium sorbate can increase the proportion of polar molecules in the films, which have a high affinity for water and, consequently, increase the water solubility of the films. However, in this study, the potassium sorbate poorly dissolved (or did not dissolve) in the film solution, which possibly interfered with the filtration of the film solutions prior to determining the solubility. Thus, the water solubility decreased.

The WVP of the films increased with increasing potassium sorbate content because adding potassium sorbate increased the polarity of the films; thus, the films absorbed more water and this increased the WVP. However, there were no significant differences (*p* > .05) in the WVP of the films, irrespective of the potassium sorbate content.

From the aforementioned findings of this study, the flour films produced with glycerol and sorbitol plasticizers (2:1 w/w) (40%, weight per dry weight of the waste flour) and 20% potassium sorbate (weight per weight of the film solution) were used to wrap fresh strawberries contained on a polystyrene foam tray. These films had suitable mechanical properties for wrapping fresh fruits because they could be extended well. Their average elongation at break (73.01%) was higher than that of the films prepared with glycerol:sorbitol at 3:1 and 4:1 w/w, respectively (Table 2). Therefore, the quality of the wrapped strawberries was studied during storage at 5°C, at 90% RH, for 0, 3, 6, and 9 days.

Strawberries are rich in vitamin C and antioxidants. They are very popular among consumers (Ahmed & Butt, 2014). If the quality and freshness of strawberries can be preserved before they reach consumers, this can reduce waste due to fruit damage. Table 3 presents the amounts of phenolic compounds, antioxidant activity, water‐soluble solids, and the weight loss, firmness and total microorganisms of the fresh strawberries stored without the film (control), wrapped by the film with glycerol and sorbitol (2:1 w/w), and wrapped by the film with glycerol:sorbitol (2:1 w/w) and formulated with 20% potassium sorbate, respectively. The amounts of phenolic compounds and antioxidant activity increased at day 3 of storage but they decreased on the days 6 and 9. Also, the strawberries wrapped by the films produced with 2:1 w/w glycerol:sorbitol and 20% potassium sorbate had higher amounts of phenolic compounds and antioxidant activity than the control and those wrapped by the film plasticized with 2:1 w/w glycerol and sorbitol but without potassium sorbate (*p* ≤ .05).

**Table 3:** Antioxidant activity, amounts of phenolic compounds, weight loss, total water‐soluble solids, firmness, and total microorganisms of the fresh strawberries stored unwrapped (control), wrapped by the films plasticized with glycerol and sorbitol (2:1), and wrapped by the films plasticized with glycerol and sorbitol (2:1) and formulated with 20% potassium sorbate (weight of potassium sorbate/weight of the film solution)

Analysis	Strawberries with and without films	Storage duration (days)			
		0	3	6	9
Antioxidant activity (mg Trolox equivalents/g fresh strawberries)	Films + potassium sorbate	2.96 ± 0.51^b^	4.02 ± 0.31^nsa^	3.65 ± 0.12^Ab^	2.66 ± 0.33^Ad^
	Films	2.96 ± 0.51^c^	3.89 ± 0.54^nsa^	3.34 ± 0.18^Ba^	2.05 ± 0.10^Bd^
	Control	2.96 ± 0.51^c^	4.17 ± 0.17^nsa^	2.99 ± 0.16^Cb^	1.74 ± 0.10^Bc^
Phenolic compound content (mg gallic acid equivalents/100 g fresh strawberries)	Films + potassium sorbate	250.76 ± 6.69^d^	509.95 ± 12.92^Aa^	442.74 ± 40.28^Ab^	326.12 ± 4.52^nsc^
	Films	250.76 ± 6.69^d^	480.04 ± 18.17^ABa^	399.87 ± 2.77^Bb^	323.02 ± 2.12^nsc^
	Control	250.76 ± 6.69^d^	427.76 ± 11.83^Ba^	392.37 ± 28.20^Bb^	302.05 ± 8.04^nsc^
Weight loss (%)	Films + potassium sorbate	0^b^	9.13 ± 0.25^Bc^	16.13 ± 0.03^Bb^	24.50 ± 0.08^Ba^
	Films	0^d^	11.35 ± 1.63^ABc^	18.23 ± 1.64^ABb^	26.31 ± 1.63^Ba^
	Control	0^d^	13.32 ± 1.21^Ab^	32.28 ± 8.38^Aa^	49.99 ± 10.33^Aa^
Total soluble solids (°Brix)	Films + potassium sorbate	9.83 ± 0.15^c^	9.97 ± 0.00^Cb^	10.00 ± 0.58^Ba^	11.67 ± 0.58^Ba^
	Films	9.83 ± 0.15^c^	10.53 ± 0.42^Bc^	11.50 ± 0.50^Bb^	12.67 ± 0.58^Ba^
	Control	9.83 ± 0.15^b^	12.53 ± 0.40^Ab^	13.83 ± 0.29^Aa^	14.67 ± 0.58^Aa^
Firmness (*N*)	Films + potassium sorbate	0.34 ± 0.04^a^	0.31 ± 0.05^Aab^	0.27 ± 0.05^nsb^	0.27 ± 0.06^nsb^
	Films	0.34 ± 0.04^a^	0.29 ± 0.07^Bab^	0.28 ± 0.07^nsb^	0.25 ± 0.07^nsb^
	Control	0.34 ± 0.04^a^	0.25 ± 0.05^Bb^	0.24 ± 0.06^nsb^	0.22 ± 0.03^nsb^
Total microorganisms (log CFU/g)	Films + potassium sorbate	2.65 ± 0.1^d^	2.07 ± 0.16^nsb^	4.61 ± 0.03^Ba^	4.95 ± 0.64^Ba^
	Films	2.65 ± 0.1^d^	2.04 ± 0.10^nsc^	5.81 ± 0.03^Aa^	6.07 ± 0.08^Ab^
	Control	2.65 ± 0.1^b^	2.05 ± 0.14^nsc^	3.87 ± 0.02^Cb^	4.37 ± 0.03^Ba^

Superscript letters (A–C) shown in the same column indicate significantly different values (*p* ≤ .05). ^ns^Means were not significantly different (*p* > .05) in the same column. Superscript letters (a–d) shown in the same row indicate significantly different values (*p* ≤ .05).

As shown in Table 3, when the storage time increased, the water‐soluble solids content also increased. The average water‐soluble solids of the strawberries that were not wrapped were the highest (14.67 °Brix) followed by those wrapped with the films plasticized with glycerol and sorbitol (2:1) (12.67 °Brix) and then those wrapped with the films plasticized with glycerol and sorbitol (2:1 w/w) and formulated with 20% potassium sorbate (11.67 °Brix). The water‐soluble solid content of the strawberries was relevant to their weight loss. The average weight loss of the strawberries that were not wrapped and those wrapped with the films plasticized with glycerol and sorbitol (2:1 w/w) were 49.99% and 26.31%, respectively. These values were higher than those wrapped with the films plasticized with glycerol and sorbitol (2:1 w/w) and formulated with 20% potassium sorbate having an average weight loss of 24.50%.

During storage, the average firmness of the strawberries that were not wrapped (control), those wrapped by the films plasticized with glycerol and sorbitol (2:1 w/w), and those wrapped by the films plasticized with glycerol and sorbitol (2:1) and formulated with 20% potassium sorbate were 0.22, 0.25, and 0.27 *N*, respectively. However, as shown in Table 3, these averages were not significantly different (*p* > .05).

The total microorganism count of all the strawberries increased with increasing storage duration. However, the strawberries wrapped by the film plasticized with glycerol and sorbitol (2:1 w/w) (no potassium sorbate) had the highest total microorganism count (average of 6.07 log CFU/g). However, no yeast and mold were present in any of the samples throughout the storage.

Shin, Ryu, Liu, Nock, and Watkins (2008) reported that the overall quality of strawberries (firmness, color, amounts of flavonoids, phenolic compounds, and antioxidant activity) stored at 10°C for 12 days decreased according to the storage duration. Also, Peretto et al. (2014) studied the quality (weight loss, appearance, firmness, color, water‐soluble solids, phenolic compounds, antioxidant activity, and microbial growth) of strawberries wrapped in films produced from strawberry puree and formulated with carvacrol, methyl, and cinnamate, after storage at 10°C for 10 days. The study found that the firmness and the brightness decreased, whereas the water‐soluble solids, phenolic compounds, and antioxidant activity, and the microbial growth inhibition of the studied strawberries, increased throughout storage.

According to the data shown in Table 3, the films effectively improved the quality of the strawberries based on the contents of phenolic compounds and antioxidant activity, and the firmness during storage (5°C, at 90% RH, for 9 days). Among the samples, the strawberries wrapped with the films produced from the waste flour plasticized with 40% glycerol and sorbitol (2:1 w/w) and formulated with 20% potassium sorbate were found to have a higher content of phenolic compounds and antioxidant activity and a higher firmness. Moreover, the strawberries wrapped by these films had a lower average total microorganism count (4.95 log CFU/g) than those wrapped by films without potassium sorbate. The Japanese local government (James, 2006), India, and several other countries (European Union, 2005, 2007; FSSAI, 2006; Mritunjay & Kumar, 2017) suggested that the total microbial plate count in safe food should be less than 5.0 log CFU/g. It can be observed from Table 3 that the microbial count found in the strawberry samples wrapped with film formulated with potassium sorbate was in the range of 2.07– 4.95 log CFU/g, indicating acceptable level for human consumption during the 9 days of storage time.

In this study, it is noticed that the films started to degrade on the 18th day (at storage temperature range of 5–45°C and 58% RH). Meanwhile, the films started to degrade after the 9th day at storage temperature of 5°C and 90% RH for wrapping fresh strawberries. From the results, it is possible to further apply the films at storage temperature range of 5–45°C and low % RH. However, this useful data from this study will be further used to enhance physico‐mechanical properties of waste flour films which can be stored at low temperature and high % RH.

## 4 CONCLUSIONS

The equilibrium moisture content of the films produced from the waste flour mixed with 75% distilled water (w/v), plasticized with 40% glycerol and sorbitol (2:1, 3:1, and 4:1 w/w) and formulated with 200 ppm potassium sorbate, was influenced by the storage temperature (5, 25, and 45°C). Also, the water activities at 0.11, 0.23, 0.32, 0.44, 0.58, 0.75, 0.84, and 0.90 were most adequately described by Henderson's equation, which obtained the highest $R_{\mathrm{adj}}^{2}$ and the lowest RMSE value, among the five mathematical model equations evaluated. Moreover, the WVP, tensile strength, and the elongation at break of the films significantly decreased (*p* ≥ .05) after storage at 58% RH, at 5, 25, and 45°C, respectively, for 30 days.

Among the films produced, those plasticized with glycerol and sorbitol (2:1 w/w) and formulated with 20% potassium sorbate had a higher average elongation at break (73.01%). Throughout the storage (5°C, 90% RH, for 9 days) of the wrapped fresh strawberries, these films maintained the quality attributes (firmness and contents of antioxidant activity and phenolic compounds) of the fruit better than the films that were not formulated with potassium sorbate. Also, the fresh strawberries wrapped by the films formulated with potassium sorbate had a lower total microorganism count than those wrapped by the films without potassium sorbate.

## CONFLICT OF INTEREST

The authors have no conflict of interest to declare.
